# ZHX1 Promotes the Proliferation, Migration and Invasion of Cholangiocarcinoma Cells

**DOI:** 10.1371/journal.pone.0165516

**Published:** 2016-11-11

**Authors:** Ryuk-Jun Kwon, Myoung-Eun Han, Ji-young Kim, Liangwen Liu, Yun-Hak Kim, Jin-Sup Jung, Sae-Ock Oh

**Affiliations:** 1 Department of Anatomy, School of Medicine, Pusan National University, Busandaehak-ro 49, Mulgeum-eup, Yangsan, 50612, Republic of Korea; 2 Gene & Therapy Research Center for Vessel-associated Diseases, Pusan National University, Busandaehak-ro 49, Mulgeum-eup, Yangsan, 50612, Republic of Korea; 3 Department of Physiology, School of Medicine, Pusan National University, Busandaehak-ro 49, Mulgeum-eup, Yangsan, 50612, Republic of Korea; University of Hong Kong, HONG KONG

## Abstract

Zinc-fingers and homeoboxes 1 (ZHX1) is a transcription repressor that has been associated with the progressions of hepatocellular carcinoma, gastric cancer, and breast cancer. However, the functional roles of ZHX1 in cholangiocarcinoma (CCA) have not been determined. We investigated the expression and roles of ZHX1 during the proliferation, migration, and invasion of CCA cells. *In silico* analysis and immunohistochemical studies showed amplification and overexpression of ZHX1 in CCA tissues. Furthermore, ZHX1 knockdown using specific siRNAs decreased CCA cell proliferation, migration, and invasion, whereas ZHX1 overexpression promoted all three characteristics. In addition, results suggested EGR1 might partially mediate the effect of ZHX1 on the proliferation of CCA cells. Taken together, these results show ZHX1 promotes CCA cell proliferation, migration, and invasion, and present ZHX1 as a potential target for the treatment of CCA.

## Introduction

Cholangiocarcinoma (CCA) is a malignant tumor arising from biliary epithelial cells, and is the sixth leading cause of gastrointestinal cancer in the West and presents a high incidence rate in East Asia [1, 2]. Furthermore, CCA mortality rates have increased worldwide over several decades. Clinical features of the disease are determined by location and clinical stage. CCAs are divided by location from the surgical perspective into intrahepatic and extrahepatic types [3, 4]. On the other hand, clinical staging which is essential for treatment and prognosis [5], depends on size, lymph node invasion, and metastasis to other tissues. No specific symptoms are observed during early stage disease and no specific early stage markers have been identified [6], and thus, CCA is usually detected in the late stage. In common with some other cancers, late detection limits the likelihood of complete tumor resection, and compromises the effectiveness of therapeutic treatments because cancer cells have already invaded lymph nodes and other tissues [7]. Accordingly, the identification of molecular targets related to the migration and invasion of CCA is of considerable therapeutic and prognostic importance.

The zinc-fingers and homeoboxes (ZHX) family consists of three proteins, ZHX1, ZHX2, and ZHX3. All members of this family contains two Cys2-His2 zinc finger motifs and five homeobox DNA-binding domains [8]. Furthermore, the homeodomain in this family is specific to vertebrate lineage. All three ZHX proteins are associated with hematopoietic cell differentiation, glomerular diseases, and hepatocellular carcinoma [9–11]. ZHX1 was firstly identified in a mouse bone marrow stromal cell line, and found to be expressed at moderate levels in lungs, spleen, and testes, and at low levels in liver and kidneys [12]. ZHX1 is composed of 873 amino acid residues and is known to repress transcription. It has several domains including two zinc finger domains at its N-terminal, five homeodomains, a nuclear localization signal domain, and an acidic region at its C-terminal [13]. ZHX1 interacts with the activation domains of NF-YA, BS69, and DNMT3B [14–16], and possible association with the progression of various cancers has also been suggested in previous studies. ZHX1 has been shown to decrease the proliferation and migration of gastric cancer cells [17], and its overexpression has been reported to reduce hepatocarcinoma cell proliferation (SMMC-7721 cells) [11]. On the other hand, its overexpression in malignant breast cancer has been associated with cancer cell invasion [18, 19]. However, the involvement of ZHX1 in the proliferation and invasiveness of CCA has not been characterized.

In the present study, we examined ZHX1 expressions in the tissues of patients with CCA, and investigated its biological effects on the proliferation, migration, and invasion of CCA cells.

## Materials and Methods

### Data analysis

The cBioPortal online platform (http://www.cbioportal.org/) includes cancer datasets released from The Cancer Genome Atlas (TCGA) database. Genomic data integrated by cBioPortal includes DNA copy-number alteration (CNAs), mRNA, and microRNA expression, and DNA methylation [20]. We used the cBioportal platform to analyze TCGA provisional datasets of liver (n = 193), breast (n = 963), pancreatic (n = 145), gastric (n = 287), and colorectal cancer (n = 220), lung squamous cell carcinoma (n = 178), cholangiocarcinoma (n = 35), kidney renal clear cell carcinoma (n = 415) and acute myeloid leukemia (n = 188). The analytical platform automatically calculated values of ZHX1 gene amplification from data based on GISTIC2 algorithm. We plotted ZHX1 gene amplification frequency in different cancers. Using cholangiocarcinoma data of cBioportal platform, ZHX1 gene amplification versus mRNA expression were also plotted. To examine whether mRNA expression of ZHX1 in CCA is higher than that in normal tissues, NCBI Gene Expression Omnibus database (GSE32225) was used.

### Immunohistochemistry

Cholangiocarcinoma tissues were obtained with written informed consent from patients who underwent surgical resection at Pusan National University Hospital and Pusan National University Yangsan Hospital. The study was approved by Pusan National University Hospital-Institutional Review Board (PNUH-IRB). Histopathologic diagnoses of CCA were performed at the Department of Pathology, Pusan National University Hospital. Tissues were deparaffinized, rehydrated, and washed using xylene, ethanol, and PBS, respectively, and then antigen retrieval was performed using the citrate buffer as previously described [21]. Endogenous peroxidase activity was inactivated with 3% hydrogen peroxide for 15min. After blocking with 1% BSA for 30 min, tissues were then treated with primary antibody, anti-ZHX1(1:75; Protein Tech, Rosemont, IL, USA) at 4°C overnight, and then with horseradish peroxidase-conjugated secondary antibody at room temperature for 40 min. Tissues were then stained with DAB (1:50) and counterstained with hematoxylin at room temperature for 3 min. Tissues were then dehydrated, washed, and mounted and examined under a light microscope at 200X. The percentage of ZHX1 positive cells in given tissues was graded on a scale of 1–4 (1, <25%; 2, 25–49%; 3, 50–74%; 4, 75–100%). The intensity of ZHX1 staining cells was decided relatively to that observed in adjacent hepatocytes (0, 1, 2, 3 for basal, weak, moderate, and strong). The overall staining score was determined by a composite score (product of the above 2 scores, ranging from 0 to 12).

### Cell culture

The HuCCT1 cell line was purchased from the Health Science Research Resources Bank (Osaka, Japan), whereas other cholangiocarcinoma cell lines (SNU245, SNU308, SNU478, SNU869, SNU1079, and SNU1196) were purchased from the Korean Cell Line Bank (Seoul, Korea). The cell lines were grown in RPMI1640 supplemented with 10% fetal bovine serum (FBS), 25mM HEPES, and 100 μg/ml of penicillin/streptomycin in a 5% CO_2_ atmosphere at 37°C.

### siRNA transfection and generation of a stable cell line

Scrambled siRNA and ZHX1 siRNA were purchased from Dharmacon (Lafayette, CO, USA) and Bioneer (Daejeon, Korea). The siRNA sequences used were as follows: ZHX1 siRNA, 5’- CUGACUUUUGAUGGUAGUU(dTdT) -3’, 5’- GAAAGUAAUGCAGGUAGUU(dTdT) -3’, 5’- CAGUUCAUCAUAACUCAGU(dTdT) -3’, scrambled siRNA (SCR), 5’- GAUCCGCAAAAGAGCGAAA(dTdT) -3’; For siRNA transfection, cholangiocarcinoma cell lines were plated at 1,500 cells/well in 96 well plate or 80,000 cells/well in 6 well plate. Cells were transfected with 100 nM of SCR or ZHX1 siRNA using DhamaFECT 1 (Thermo Scientific, Kafayette, CO, USA), within 1 day of plating cells, according to the manufacturer’s instructions. To assure the efficiency of ZHX1 knockdown for proliferation, migration and invasion assays, we assigned samples to check the efficiency for each experiment. When the mRNA level of ZHX1 was decreased by more than 80%, the results were included for the further analysis. In order to prepare ZHX1 expression vector, the ZHX1 gene (Invitrogen, Dane, WI, USA) was inserted into 3XFlag CMV-10 vector (E4401; Sigma-Aldrich Korea, Seoul, Korea). To generate a stable cell line, HuCCT1 cells were plated at 200,000 cells/well in 6 well plate and transfected with empty vector or the vector containing the ZHX1 gene using ViaFect^TM^ (Promega, Madison, WI, USA), according to the manufacturer’s instructions. G418 (500 μg/ml; Roche Diagnostics GmbH, Mannheim, Germany) was used to select ZHX1-overexpressing clones.

### Tissue and Real-time PCR

The gallbladder tissues used for determination of ZHX1 expression were provided by the Biobank of Pusan National University Hospital, a member of the Korea Biobank Network. The tissues were obtained with informed consent from patients who underwent surgical resection at Pusan National University Hospital and the present study was approved by the institutional review board of the hospital (PNUH-IRB). Total RNAs were extracted using the RNeasy Mini kit (Qiagen, Valencia, CA, USA). Purified RNA were used to synthesize cDNA using oligo-dT, dNTP, RNasin, MMLV reverse transcriptase (Promega, Madison, WI, USA). cDNAs obtained were used as templates for real-time PCR. cDNAs were mixed with FastStart Essential DNA Green Master (Roche Diagnostics GmbH, Mannheim, Germany) to determine ZHX1 mRNA levels. Real-time PCR and analysis were performed on a LightCycler^TM^ 96 Real-time PCR system (Roche, Nonnenwald, Germany). Three separate experiments were performed and GAPDH was used as the internal control. The primer sequences for real-time PCR were as follows: GAPDH forward primer 5’- TGG TGA CCA GGC GCC CAA TAC G -3’, GAPDH reverse primer 5’- GCA GCC TCC CGC TTC GCT CT -3’, ZHX1 forward primer 5’- TCC CTT ACC CAA CAA TGT CA- 3’, ZHX1 reverse primer 5’- TTG TTT CCT TCT TGC CTC CT -3’, EGR1 forward primer 5’- CTT TTC CCT GGA GCC TGC AC -3’, EGR1 reverse primer 5’- AAT GTC AGT GTT CGG CGT GG -3’.

### Western blotting

Proteins were prepared from cells lysed with RIPA buffer (Thermo Scientific, Rockford, IL, USA) containing protease inhibitor cocktail and β-mercaptoethanol. Protein concentrations were determined using the Bio-Rad protein assay kit (Bio-Rad, Hercules, CA, USA). Protein samples (20 or 40 μg) were separated by 8% acrylamide gel SDS-PAGE and then transferred to nitrocellulose membranes (Amersham Biosciences UK Ltd, Buckinghamshire, UK) at 4°C overnight. Membranes were blocked in 5% nonfat dry milk for 1 hour and incubated with antibodies diluted in PBS (phosphate buffered saline) containing 5% BSA at 4°C overnight, and then treated with secondary antibody, horseradish peroxidase-conjugated anti-mouse, or anti-rabbit antibody at room temperature for 1 hour. A chemiluminescent agent (EZ-Western Detection Kit, Seoul, Korea) was applied to detect protein bands using the LAS-3000 imaging system (Fujifilm, Tokyo, Japan), and quantification was performed using ImageJ software. Three separate experiments were performed and β-actin was used as the internal control. The antibodies used for western blotting were as follows; anti-ZHX1 (1:1000; Protein Tech, Chicago, IL, USA), and anti-β-actin (1:5000; Santa Cruz Biotechnology, Dallas, TX, USA)

### Cell proliferation assay

To examine CCA cell proliferation, we used Ez-Cytox proliferation assay kit (ITSBIO, Seoul, Korea) according to the manufacturer’s instructions. In brief, 10 μl of Ez-Cytox was added to wells 3 to 5 days after SCR or ZHX1 siRNA transfection and after plating Mock or ZHX1-over cells. Cells were then placed in CO_2_ incubator for 1 to 2 hours, and proliferation was measured at 450 nm using an ELISA reader (TECAN, Mannedorf, Switzerland). SCR or Mock values were used as controls to calculate relative cell proliferation. Experiments were performed in triplicate.

### Boyden chamber assay

To examine CCA cell migration, 48-well micro chemotaxis chambers (Neuro Probe, Gaithersburg, MD, USA) were used. Culture medium containing 10% FBS (50 μl) was added to bottom chambers, which were then covered with collagen coated membranes (Neuro Probe, Gaithersburg, MD, USA). For collagen coating, 5 ml of PBS containing 0.02 μg/μl of collagen was treated for 30 min. Cells (5x10^4^) in 50 μl of medium containing 0.05% FBS were seeded in upper chambers. After 5 to 10 hours, cells that migrated through membrane were prepared by fixing and then staining membranes using Diff-quik solution (Sysmex, Kobe, Japan). The stained membranes were washed and were attached on micro slide glasses (Matsunami glass, Osaka, Japan). Experiments were performed in triplicate. Pictures were taken using a light microscope at 40X and 200X. Total numbers of migrated cells were counted using Adobe Photoshop CS6 software. SCR or Mock values were used as controls to calculate relative cell migration.

### Wound healing assay

Mock cells or ZHX1-over cells were seeded at 7.5x10^4^ per well in 6-well plates. Cells were scratched one day after seeding, and then fresh medium containing 100 ng/ml of mitomycin C and 0.1% FBS was added. Pictures were taken immediately and 21hr after scratching. Five separate experiments were performed. For quantification purposes, scratch widths were measured. Results were normalized by the results of Mock-treated controls.

### Matrigel invasion assay

24-well BioCoatTM Matrigel chamber inserts (BD Bioscience, San Jose, CA, USA) were used for this experiment. The upper surfaces of invasion chambers were coated with 30 μl of 0.5 mg/ml growth factor-reduced Matrigel (BD Bioscience, San Jose, CA, USA), and lower surfaces were coated with 20 μl of 0.5 mg/ml fibronectin (Sigma-Aldrich Korea, Seoul, Korea). Before starting the experiment, coated inserts were rehydrated with RPMI1640 for 4 hours in a humidified 5% CO_2_ incubator. Cells (5x10^4^) in 500 μl of media containing 0.1% FBS were plated into invasion chambers 36 hours after SCR or ZHX1 siRNA transfection. RPMI1640 medium containing 10% FBS (700 μl) was added to each well as a chemoattractant. Mitomycin C (Sigma-Aldrich Korea, Seoul, Korea) was used to inhibit cholangiocarcinoma cell proliferation. After incubation for 18 or 80 hours, non-invading cells on upper insert surfaces were removed cleaned by scraping. Invaded cells in inserts were fixed and stained using Diff-quik solution (Sysmex, Kobe, Japan). Experiments were performed in triplicate. Pictures were taken under a light microscope at 20x and 56x. Total numbers of invaded cells were counted using Adobe Photoshop CS6 software.

### Pathway Reporter Array

To perform a screening test for identification of targets regulated by ZHX1, the Cignal Finder 45-Pathway Reporter Array (Qiagen, Dusseldorf, Germany) was used. Complex formation mixed with Opti-MEM and Attractene was first prepared, according to the manufacturer’s instructions. 50 μl of suspended cells (8×10^5^ cells/ml) were added to each well containing the prepared complex and were incubated in a 5% CO_2_ incubator at 37°C. After two days, the luciferase reporter assays were performed.

### Statistical analysis

The Mann-Whitney U-test or the Student’s t-test were used to determine the significances of differences between the mean values of two groups. * and ** indicates a P value of <0.05 and <0.01, respectively, which was regarded the significance criterion. Data were analyzed using SPSS software, version 12.0 (SPSS Inc., Chicago, IL, USA). Results are presented as means±SEs.

## Results

### Expressions of ZHX1 in cholangiocarcinoma (CCA) tissues and cell lines

**Fig 1:**
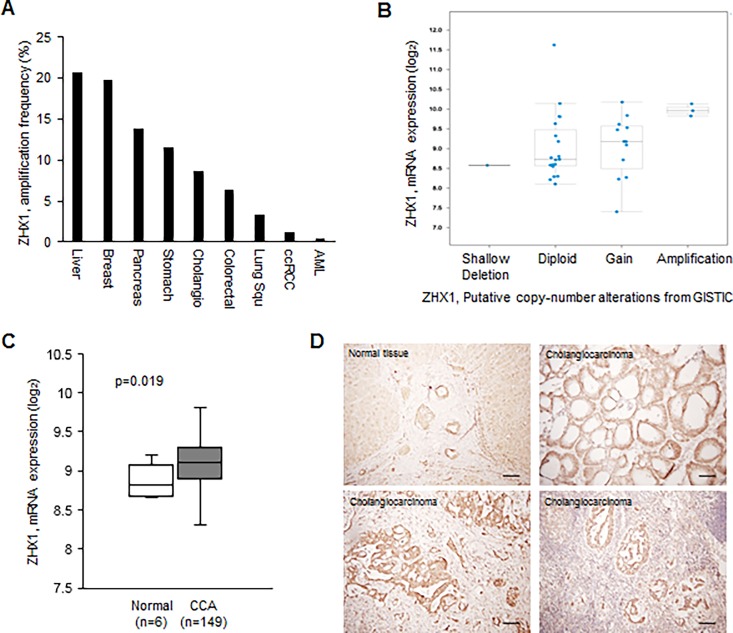
ZHX1 was overexpressed in cholangiocarcinoma (CCA). (A) ZHX1 amplification frequency was analyzed in various cancers using TCGA data. ZHX1 gene was found to be amplified in various cancers including CCA. Cholangio (Cholangiocarcinoma), Colorectal (Colorectal adenocarcinoma), Lung Squ (Lung squamous cell carcinoma), ccRCC (kidney renal clear cell carcinoma), AML (acute myeloid leukemia) (B) Using cBioPortal online platform, the correlation between ZHX1 gene amplification and expression of ZHX1 mRNA was plotted. (C) Using NCBI Gene Expression Omnibus database (GSE32225), the ZHX1 expression in normal bile duct and cholangiocarcinoma tissues was analyzed. (D) Immunohistochemistry showed ZHX1 was overexpressed in some CCA tissues (N = 10), and notably, ZHX1 overexpression was observed in CCA cells that had invaded lymph nodes.

We first analyzed ZHX1 gene alteration in various cancers using TCGA data. *In silico* analysis showed that ZHX1 is amplified in many cancers, including breast cancer, gastric cancer, and CCA (Fig 1A). To examine whether the amplification of ZHX1 gene in CCA are associated with the upregulation of ZHX1 mRNA, cBioPortal online platform was used. The mRNA expression level of ZHX1 gradually increased as its copy number increased (Fig 1B). This result shows that the amplification of ZHX1 gene is associated with its overexpression in CCA. Next, to determine whether ZHX1 expression is higher in CCA than in normal tissues, we compared the mRNA expression levels of ZHX1 in CCA with those in normal tissues using NCBI Gene Expression Omnibus database (GSE32225). The analysis showed that ZHX1 was found to be overexpressed in CCA tissues versus normal tissues (Fig 1C). Moreover, we compared ZHX1 expressions of CCA cell lines (HuCCT1, SNU308, SNU478, SNU1079, SNU1196) with those of normal gallbladder tissues using real-time PCR and western blotting. The reason why we used gallbladder tissues as a control is that it is hard to obtain normal bile duct tissues and the histological structure of gallbladder tissue is similar to that of bile duct tissue [22]. ZHX1 expressions in the CCA cell lines were higher than those in normal gallbladder tissues (S1 Fig). This observation is interesting because ZHX2 has been reported to be down-regulated in hepatocellular carcinoma [11] and multiple myeloma [23]. To further confirm whether ZHX1 is overexpressed in CCA, we examined the expressional status of ZHX1 in patients’ CCA tissues using immunohistochemistry, which was performed on 4-μm paraffin-embedded sections. ZHX1 was found to be highly expressed in some CCA tissues versus normal tissues (N = 10, 7/10, Fig 1D). Interestingly, ZHX1 was overexpressed in CCA cells that had invaded lymph nodes (Fig 1D).

### A role of ZHX1 in the proliferation of CCA cells

**Fig 2:**
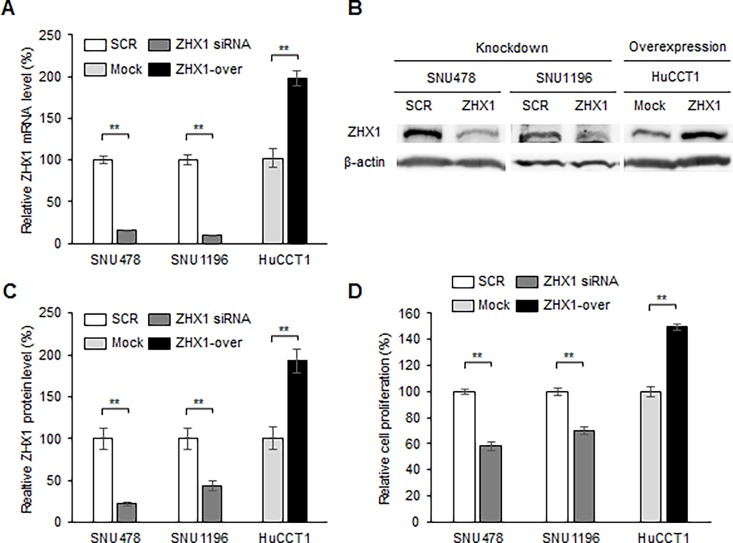
ZHX1 regulated the proliferation of CCA cells. (A) The effect of knockdown or overexpression of ZHX1 on the level of mRNA was examined by real-time PCR. For ZHX1 knockdown, CCA cells were treated with 100 nM of scrambled siRNA (SCR) or ZHX1 siRNA. SCR siRNA-treated samples were used as controls. For the gain-of-function study, ZHX1-overexpressing CCA cells (ZHX1-over) were generated from HuCCT1 cells, and empty control vector-expressing (Mock) cells were used as a control. GAPDH was used as an internal control. (B) Knockdown and overexpression efficiencies were determined by western blotting. (C) ZHX1 protein levels were quantified using image J software, and β-actin was used as an internal control. SCR siRNA-treated controls in the knockdown study, and Mock cells in the gain-of-function study were used as controls. (D) Cell proliferation was measured 3 to 5 days after SCR or ZHX1 siRNA transfection. SCR siRNA-treated controls in the knockdown study and Mock cells in the gain-of-function study were used as controls to calculate relative cell proliferation. Bar graphs show the means ± SEs of three independent experiments. *, P < 0.05, **, P<0.01, versus SCR or Mock.

The overexpression of ZHX1 in CCA suggests it has oncogenic roles, and thus, we examined its effects on the characteristics of CCA cells by its knockdown and overexpression. The efficiency of ZHX1 knockdown by specific siRNA was determined by real-time PCR and western blotting. The mRNA levels of ZHX1 in SNU478 and SNU1196 cells were reduced by ZHX1 siRNA versus scrambled siRNA by 84.8% and 90.2%, respectively (Fig 2A), and the ZHX1 protein levels in SNU478 and SNU1196 were reduced by ZHX1 siRNA versus scrambled siRNA by 78.1% and 56.1%, respectively (Fig 2B and 2C). We also generated a ZHX1-overexpressing stable cell line from HuCCT1. The mRNA and protein levels of ZHX1 were found to be higher in ZHX1-overexpressing HuCCT1 cells than Mock cells (empty control vector-expressing cells) by 198.4% and 192.7%, respectively (Fig 2A–2C). We next examined the proliferation of ZHX1 knocked-down and ZHX1-overexpressing cells. Cell proliferation assays were performed from 3 to 5 days after SCR or ZHX1 siRNA transfection and after plating Mock or ZHX1-overexpressing HuCCT1 cells. The proliferation of SNU478 and SNU1196 cells were significantly reduced by ZHX1 siRNA versus SCR siRNA by 41.8% and 29.9% respectively (Fig 2D). A similar result was obtained in HuCCT1 cells (S2 Fig). In contrast, the proliferation of ZHX1-overexpressing HuCCT1 cells was significantly greater than that of Mock cells by 149.5% (Fig 2D). A similar result was also obtained in ZHX1-overexpressing SNU478 cells (S2 Fig). These results indicate that ZHX1 promotes the proliferation of CCA cells.

### Roles of ZHX1 in migration and invasion of cholangiocarcinoma cells

**Fig 3:**
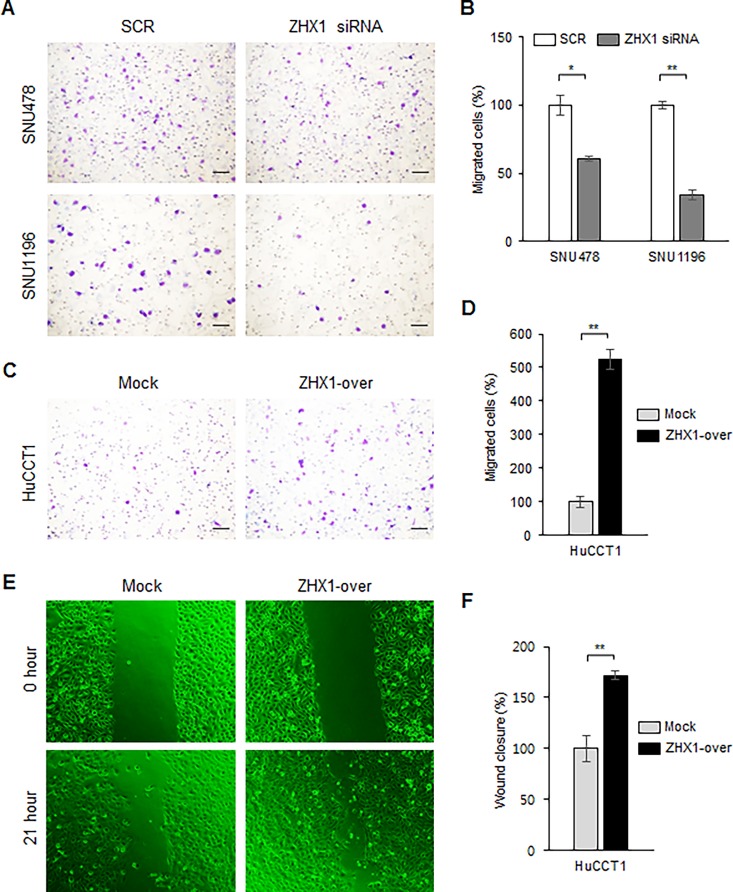
ZHX1 promoted the migration of cholangiocarcinoma cells. Migration was examined using a Boyden chamber assay and a wound healing assay. (A) ZHX1 siRNA significantly inhibited the FBS-induced migrations of SNU478 and SNU1196 cells compared with SCR siRNA. Scale bar represents 50 μm. (B) The number of migrated cells were counted and SCR siRNA-treated controls were used as controls to calculate the percentage of cell migration in ZHX1-knockdown cells. The values are shown as the bar graph. Results are the means ± SEs of three independent experiments. (C) Overexpression of ZHX1 significantly increased the migration of HuCCT1 cells versus Mock cells. Scale bar represents 50 μm. (D) The number of migrated cells were counted and Mock cells were used as controls to calculate the percentage of cell migration in ZHX1-overexpressing cells. Results are the means ± SEs of three independent experiments. (E) Migration of cholangiocarcinoma cells was examined using a wound healing assay. Cells were scratched one day after seeding, washed twice with 1X PBS, and then the fresh media containing 100 ng/ml of mitomycin C and 0.1% FBS was added. Pictures were taken at 0 hour and 21 hours after scratching. Five separate experiments were performed. (F) Migration was quantified by measuring the scratch widths. Mock cells were used as controls. The bar graphs show the means ± SEs of five independent experiments. *, P < 0.05, **, P<0.01, versus SCR or Mock.

To examine effects of ZHX1 on CCA cell migration, we used a Boyden chamber assay two days after SCR or ZHX1 siRNA transfection and after seeding Mock or ZHX1-overexpressing HuCCT1 cells. The results showed that fewer SNU1196 and SNU478 cells treated with ZHX1 siRNA migrated than cells treated with SCR siRNA (Fig 3A). The migrations of SNU478 and SNU1196 cells were significantly reduced by ZHX1 siRNA versus SCR siRNA by 39% and 66%, respectively (Fig 3B). A similar result was observed in HuCCT1 cells (S2 Fig). In contrast, significantly more (524%) ZHX1-overexpressing HuCCT1 cells migrated than Mock cells (Fig 3C and 3D). A similar result was also observed in ZHX1-overexpressing SNU478 cells (S2 Fig). A wound healing assay was used to confirm the effects of ZHX1 on CCA cell migration. To eliminate proliferation-associated effects, we used the culture medium containing 100 ng/ml of mitomycin C and 0.1% FBS. Scratch widths were used to quantify results. Results showed that the migration of ZHX1-overexpressing HuCCT1 cells was significantly greater than that shown by Mock cells by 171.9% (Fig 3E and 3F).

**Fig 4:**
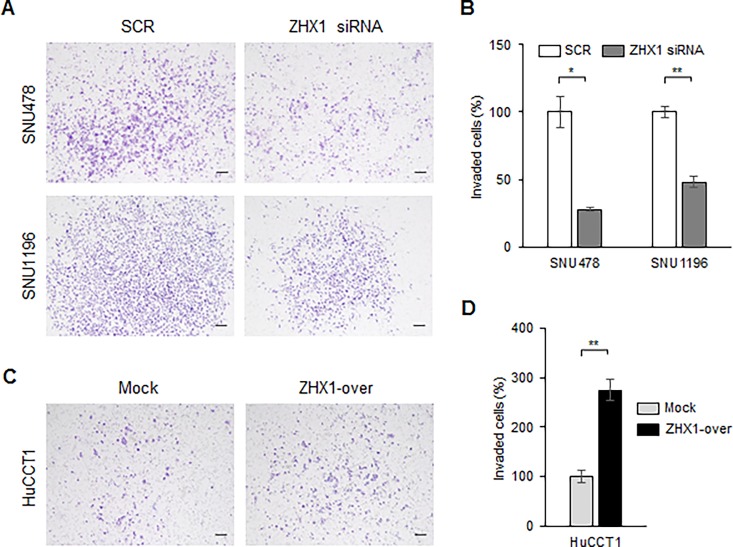
ZHX1 accelerated the invasion of cholangiocarcinoma cells. A matrigel invasion assay was used to examine the invasive ability of CCA cells. (A) ZHX1 siRNA decreased the FBS-induced invasion of SNU478 and SNU1196 cells compared with SCR siRNA. (B) The numbers of invaded cells were counted and SCR siRNA-treated cells were used as controls to calculate the percentage of cell invasion. Results are the means ± SEs of three independent experiments. (C) Overexpression of ZHX1 significantly increased the invasion of HuCCT1 cells versus Mock cells. Scale bar represents 50 μm. (D) The numbers of invaded cells were counted and Mock cells were used as controls to calculate the percentage of cell invasion. Results are the means ± SEs of three independent experiments. *, P < 0.05, **, P<0.01, versus SCR or Mock.

Next, we examined the effects of ZHX1 on CCA cell invasion using matrigel-coated inserts. Knockdown of ZHX1 inhibited the invasion of SNU478 and SNU1196 cells as compared with SCR cells by 72% and 52%, respectively (Fig 4A and 4B). In contrast, ZHX1-overexpressing HuCCT1 cells had greater invasion rates than Mock cells by 278.7% (Fig 4C and 4D). These results indicated ZHX1 plays important roles in the migration and invasion of CCA cells.

### Regulation of EGR1 by ZHX1

**Fig 5:**
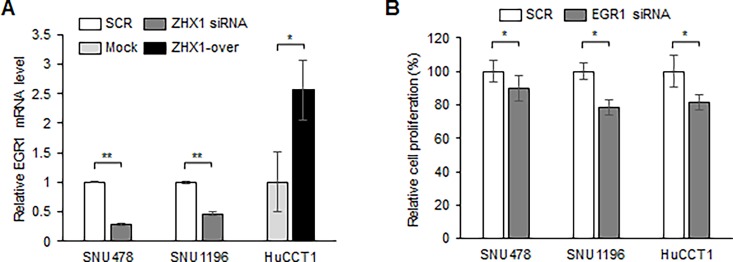
ZHX1 regulated EGR1 expression in cholangiocarcinoma cells. (a) Real-time PCR was used to assess EGR1 mRNA level changes. EGR1 mRNA levels were measured two days after SCR or ZHX1 siRNA transfection in SNU478 or SNU1196 cells and after plating Mock and ZHX1-overexpressing HuCCT1 cells. (b) Cell proliferation was observed 3 to 4 days after SCR or EGR1 siRNA transfection in SNU478, SNU1196 and HuCCT1 cells. SCR values were used as controls to calculate relative cell proliferations. Results are presented in the bar graphs as the means ± SEs of three independent experiments. *, P < 0.05, **, P<0.01, versus SCR or Mock.

To identify the mechanism responsible for the promotion of proliferation, migration, and invasion of CCA cells by ZHX1, we performed a preliminary screening test using the Cignal Finder 45-Pathway Reporter Array (S3 Fig). We found that HSF1, c-MYC, OCT4, and EGR1 were upregulated and C/EBP was downregulated by ZHX1 overexpression. Because the Reporter Array measures only the luciferase activity of various transcription factors, to further confirm the Reporter Array results, we measured the mRNA expression of each transcription factor using real time PCR after knockdown and overexpression of ZHX1 (data not shown). Among tested transcription factors, EGR1 was regulated by ZHX1 consistently with the Reporter Array results (Fig 5A). Interestingly, the EGR1 gene has been previously shown to be associated with the progression of cholangiocarcinoma [24, 25]. Confirmatory real-time PCR showed ZHX1 knockdown decreased EGR1 expression in SNU478 and SNU1196 cells (Fig 5A), whereas ZHX1 overexpression increased EGR1 mRNA levels (Fig 5A). Subsequently, EGR1 knockdown was found to reduce the proliferations of SNU478, SNU1196 and HuCCT1 cells (Fig 5B). Because the reduction in the proliferation of SNU478 cells by EGR1 siRNA is less than that by ZHX1 siRNA, other molecules might be involved in the action of ZHX1. These observations suggest ZHX1 regulates the proliferation of CCA cells partially by controlling EGR1 expression.

## Discussion

ZHX1 is a novel transcription factor and was recently discovered in a mouse bone marrow stromal cell line, and thus, comparatively little is known of the pathophysiological roles of ZHX1 during cancer progression. Here, we report for the first time ZHX1 promotes the proliferation, migration, and invasion of CCA cells, and that it upregulates EGR1, which has been previously shown to promote progression of various cancers including prostate and ovarian cancer. All results for effects of ZHX1 on cholangiocarcinoma cells were summarized as a table (S1 Table).

The roles played by ZHX family members in proliferation was first revealed by studies on ZHX2, which was found to suppress the proliferation of hepatocellular carcinoma cells by inducing cell cycle arrest at G1 [26]. In Hodgkin’s lymphoma, ZHX2 expression was diminished and ZHX2 knockdown suppressed genes promoting differentiation and apoptosis [27]. Furthermore, it was recently reported ZHX1 inhibited gastric cancer cell proliferation [17], and that ectopic ZHX1 expression decreased proliferation of SMMC-7721 cells (a hepatocellular carcinoma cell line) [11]. However, other studies have shown ZHX1 promotes cell proliferation. For example, in CTLL-2 cells (cytotoxic T cells), IL-2 increased ZHX1 gene expression and CTLL-2 proliferation [28], and in malignant breast cancer, an elevated ZHX1 level was associated with cancer cell invasion [18, 19]. In addition, in Hodgkin’s lymphoma, ZHX1 was found to inhibit B-cell differentiation and to be associated with the pathogenesis of lymphoid malignancy [9]. Similarly, in the present study, ZHX1 knockdown reduced and its overexpression increased cell proliferation in cholangiocarcinoma cell lines (Fig 2 and S2 Fig). These results indicate that effect of ZHX1 on cancer cell proliferation is cell type-specific. Cell type-specific roles of a specific pathway has been demonstrated in various signaling pathways including hedgehog signaling pathway [29].

The present study also provides evidence ZHX1 positively regulates the motility and invasiveness of cholangiocarcinoma cells. CCA cell migration and invasion were suppressed by ZHX1 knockdown and increased by ZHX1 overexpression in our Boyden chamber assay and matrigel invasion assay, and ZHX1-over cells migrated faster than Mock cells in the wound healing assay (Figs 3 and 4, S2 Fig). In addition, our IHC investigation of CCA tissues showed that ZHX1 was highly expressed in invaded lymph nodes (Fig 1C). These results imply ZHX1 is required for migration and invasion during CCA progression. Consistent with our results, in non-metastatic human breast cancer cells, ZHX1 expression was reduced by Pea3 knockdown, and this led to decreases in invasiveness and metastasis [18].

ZHX1 expression has been reported to be decreased in hepatocellular carcinoma tissues [11]. However, two patients (Total N = 12) showed overexpression in that study. In the present study, the ZHX1 gene was amplified in various cancer cells, including breast cancer, gastric cancer, and CCA (Fig 1A). We also observed ZHX1 overexpression in CCA tissues (Fig 1C and 1D). Moreover, the ZHX1 mRNA level in CCA cell lines was higher than that in normal gallbladder tissues (S1 Fig). Additional studies need be undertaken to determine the nature of the relation between ZHX1 expression and prognosis.

The present study showed ZHX1 regulated the level of EGR1 (early growth response protein 1, Fig 5), which is a multifunctional transcription factor that regulates cellular processes as diverse as survival and apoptosis. EGR1 has been reported to be involved in the progression of prostate cancer and to increase invasion by ovarian cancer cells [30, 31]. The expression of EGR1 can be induced by various growth factors such as EGF and HGF and stress signals [32, 33]. Interestingly, EGF has been shown to promote the migration and invasion of CCA cells, and knockdown of thymosin β10, which leads to the increase in CCA metastasis, was found to increase EGR1 expression in CCA cells [25]. Furthermore, the expression of EGR1 was decreased by silencing PRR11, which has oncogenic effects in hilar cholangiocarcinoma [24].

In summary, we found ZHX1 was found to play essential roles in the proliferation, migration, and invasion of cholangiocarcinoma cells, and that its effect on the proliferation was mediated partially through EGR1. We hope these results contribute to the development of a new therapeutic strategy for the treatment of cholangiocarcinoma.

## Supporting Information

S1 Fig The ZHX1 expression in a normal gallbladder tissue and CCA cell lines. (A) The ZHX1 expressions of a normal gallbladder tissue and CCA cell lines (HuCCT1, SNU308, SNU478, SNU1079, SNU1196) were determined by real-time PCR. A Gallbladder tissue was used as a control because the histological structure of gallbladder and bile duct tissues is similar. (B) ZHX1 protein levels were determined by western blotting, and β-actin was used as an internal control.
(TIFF)

S2 Fig The effect of ZHX1 on the proliferation and migration of CCA cells. (A) Proliferation assay was performed 3 to 5 days after SCR or ZHX1 siRNA transfection. SCR siRNA-treated controls and Mock cells were used as controls to determine relative cell proliferation. Bar graphs show the means ± SEs of three independent experiments. *, P < 0.05, **, P<0.01, versus SCR or Mock. (B) Migration assay was performed using a Boyden chamber assay. ZHX1 siRNA treatment inhibited the FBS-induced migration of HuCCT1 cells and ZHX1 overexpression increased the FBS-induced migration of SNU478 cells. (C) Number of migrated HuCCT1 and SNU478 cells were counted. SCR siRNA-treated control and Mock cells were used as controls to determine the relative migration rates of ZHX1knockdown and overexpression cells. Results are shown as a bar graph, and are the means ± SEs of three independent experiments. *, P < 0.05, **, P<0.01, versus SCR or Mock.
(TIFF)

S3 Fig Candidate targets regulated by ZHX1. (A) To identify targets regulated by ZHX1, the Cignal Finder 45-Pathway Reporter Array was performed according to the manufacturer’s instructions. 50ul of suspended cells (8×10^5^cells/ml) were mixed with complex formation for transfection. The luciferase reporter assay was performed 2day after transfection.
(TIFF)

S1 Table Summarization for effects of ZHX1 on cholangiocarcinoma cells. (TIFF)
