# Supplementary figures and images for: ZHX1 Promotes the Proliferation, Migration and Invasion of Cholangiocarcinoma Cells
Source: PLoS One. 2016 Nov 11;11(11):e0165516. doi: 10.1371/journal.pone.0165516 (PMC5105949; doi:10.1371/journal.pone.0165516)

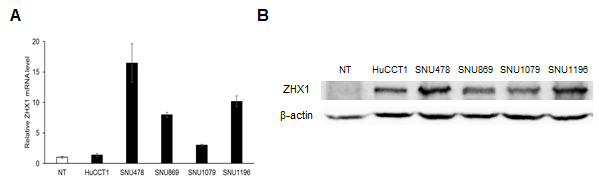

Supplement: S1 Fig — (A) The ZHX1 expressions of a normal gallbladder tissue and CCA cell lines (HuCCT1, SNU308, SNU478, SNU1079, SNU1196) were determined by real-time PCR. A Gallbladder tissue was used as a control because the histological structure of gallbladder and bile duct tissues is similar. (B) ZHX1 protein levels were determined by western blotting, and β-actin was used as an internal control. (TIFF) [file pone.0165516.s001.tiff]

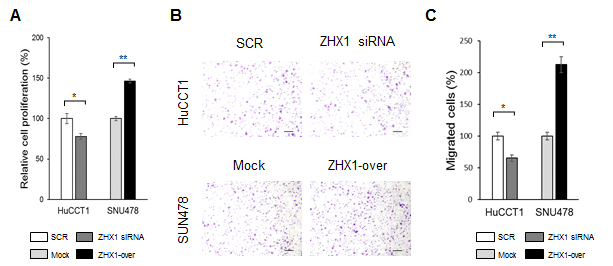

Supplement: S2 Fig — (A) Proliferation assay was performed 3 to 5 days after SCR or ZHX1 siRNA transfection. SCR siRNA-treated controls and Mock cells were used as controls to determine relative cell proliferation. Bar graphs show the means ± SEs of three independent experiments. *, P < 0.05, **, P<0.01, versus SCR or Mock. (B) Migration assay was performed using a Boyden chamber assay. ZHX1 siRNA treatment inhibited the FBS-induced migration of HuCCT1 cells and ZHX1 overexpression increased the FBS-induced migration of SNU478 cells. (C) Number of migrated HuCCT1 and SNU478 cells were counted. SCR siRNA-treated control and Mock cells were used as controls to determine the relative migration rates of ZHX1knockdown and overexpression cells. Results are shown as a bar graph, and are the means ± SEs of three independent experiments. *, P < 0.05, **, P<0.01, versus SCR or Mock. (TIFF) [file pone.0165516.s002.tiff]

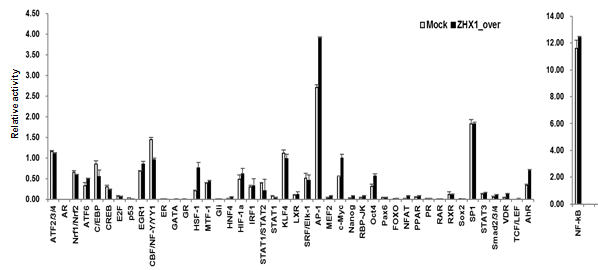

Supplement: S3 Fig — (A) To identify targets regulated by ZHX1, the Cignal Finder 45-Pathway Reporter Array was performed according to the manufacturer’s instructions. 50ul of suspended cells (8×10^5^cells/ml) were mixed with complex formation for transfection. The luciferase reporter assay was performed 2day after transfection. (TIFF) [file pone.0165516.s003.tiff]

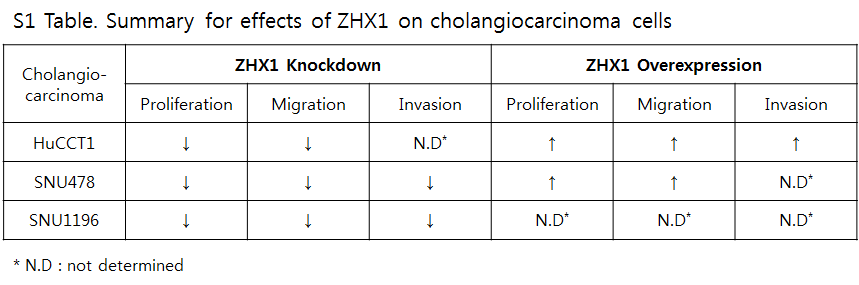

Supplement: S1 Table — (TIFF) [file pone.0165516.s004.tiff]
